# Retraction: Synergistic antitumor activities of docetaxel and octreotide associated with apoptotic-upregulation in castration-resistant prostate cancer

**DOI:** 10.1371/journal.pone.0336085

**Published:** 2025-11-05

**Authors:** The PLOS One Editors

After this article [1] was published, concerns were raised about Figs 1, 4, 5, S4 and S5.

Specifically:

- In Fig 1, panels D and E appear to partially overlap.
- The chart in Fig 4B appears similar to the chart in Fig 4F.
- In Fig 5A, the left two panels appear similar.
- In Fig S4A, when levels are adjusted to visualize the background, there appear to be vertical discontinuities in the CASP 3 and CASP 9 panels.
- Results from an animal study are reported in Fig S5, but no details on the experimental methodology, animal welfare or ethics approval are reported in the article.

The authors did not respond to editorial follow-up on these concerns.

In the absence of the original data underlying the figures of concern, the above concerns remain unresolved and the *PLOS One* Editors retract this article.

All authors either did not respond directly or could not be reached.
